# Parental Perspectives and Experiences of Working With Speech and Language Therapists to Support Home Practice for Their Child With a Speech Sound Disorder: A Qualitative Study

**DOI:** 10.1111/1460-6984.70280

**Published:** 2026-06-22

**Authors:** Katherine Pritchard, Vesna Stojanovik, Jill Titterington, Emma Pagnamenta

**Affiliations:** ^1^ School of Psychology and Clinical Language Sciences University of Reading Reading Berkshire UK; ^2^ The Speech Doctor Holywood Northern Ireland UK

**Keywords:** behaviour change, home‐practice, parents, speech sound disorder

## Abstract

**Background:**

Speech sound disorder (SSD) is broadly defined as difficulty producing speech sounds in childhood. It can have a lasting impact on academic outcomes and well‐being, making effective early intervention vital. Speech and language therapists (SLTs) consider parental involvement—particularly supporting their child with home practice—as essential to a child's progress. Relationships between SLTs and parents are known to facilitate this engagement. However, there is a significant gap in the literature regarding parents’ perspectives, and little is known about what parents perceive to be most effective in supporting home practice. Understanding these perspectives is crucial for designing interventions that are both feasible and meaningful for families.

**Aim:**

To explore the perspectives of parents of children with SSD aged ≤5;11, about their experiences with speech and language therapy intervention sessions and home practice.

**Methods and Procedures:**

This study used qualitative methodology. Nine parents, recruited via social media, professional networks and a university clinic, participated in focus groups or 1:1 interviews. A group of children, aged 4–6, who had lived experience of SSD, speech and language therapy and home practice were consulted to design the topic guide and inform data analysis. Discussions were recorded, transcribed verbatim and analysed using Reflexive Thematic Analysis.

**Outcomes and Results:**

Four main themes were constructed as follows: (1) Building positive therapeutic relationships is fundamental to families’ experiences and creates the foundation for successful home practice. (2) SLTs need to employ a wide range of skills and knowledge, including a multi‐modal approach to empower parents and develop their capability. (3) Clear communication is key for shared power, understanding of roles, active involvement and effective home practice. (4) Factors outside an individual's control can impact parents’ attitudes towards, and engagement with, home practice.

**Conclusions and Implications:**

We conclude that relationships between SLTs and parents, and the skills required to build these, underpin parental motivation to engage in home practice. To maximise parental capability, SLTs ensure that parents understand not only what to do but why they are doing it, utilising a variety of methods. Whilst some barriers for home practice are out of the SLT's control, using all opportunities to develop the motivation and capability of the parents is required. To achieve this, service delivery models may need to be reconsidered.

**WHAT THIS PAPER ADDS:**

*What is already known on this subject*

- SSD that persists into school years can have lasting implications for a child's academic outcomes and well‐being. Effective and efficient early intervention is essential. SLTs and parents agree that working together, including parental delivery of home practice, is important for a child's progress. Little is known about how parents experience and perceive what SLTs do within intervention sessions to ensure parents feel confident and capable of delivering effective home practice.

*What this paper adds to existing knowledge*

- This is the first study to explore parents’ perceptions and experiences of what SLTs do within direct intervention sessions for young children with SSD and how this supports parents to become implementors of intervention for their child at home. Findings suggest that parents benefit from SLTs using a range of teaching and coaching approaches, including clear instructions and active involvement, to ensure parents feel confident and capable at home. Relationships between the parent, SLT and the child are highly valued by parents, and are fundamental for parental engagement with home practice.

*What are the potential or actual clinical implications of this work?*

- SLTs prioritising building effective relationships with parents and children can support parental motivation for home practice. SLTs can work with parents to increase opportunities for home practice by building it into their daily lives, including working with other family members. To maximise parental capability, it is important to ensure that parents understand not only what to do but why they are doing it, utilising a variety of methods. To achieve this, service delivery models may need to be reconsidered.

## 1 Background

Speech sound disorder (SSD) is an overarching term used for difficulties with speech sounds, syllable structure and prosody that impact a child's speech clarity in childhood. It encompasses difficulties of known and unknown origin and can be further sub‐divided into phonological difficulties and motor/phonetic difficulties (Stringer et al. 2024). SSD that persists past Age 8 can have a negative impact on a child's academic outcomes, social relationships and well‐being (Wren et al. 2021; Wren et al. 2023), including an increased likelihood to report self‐harm with suicidal intent in adolescence (McAllister et al. 2023), highlighting the need for effective and efficient early intervention.

Working alongside parents as partners is recommended as best practice in the United Kingdom (HCPC 2024). Speech and language therapists (SLTs) consistently agree that working with parents to support them in implementing effective home practice is vital for a child with SSD to progress (Sugden et al. 2018; Watts Pappas et al., 2008). Parents also value home practice for their children with SSD (Sugden et al. 2019). Emerging evidence suggests that there is a conflict between SLTs’ beliefs and practice. While SLTs endorse parent involvement in decision making and intervention delivery for their children with SSD (Sugden et al. 2018; Watts Pappas et al., 2008), one study found that, in practice, intervention sessions were led by SLTs with minimal parental input (Watts Pappas et al. 2008).

The COM‐B model offers a framework for understanding how SLTs support parental engagement in intervention sessions and home practice. Within this model, **capability**, **opportunity** and **motivation** are seen as drivers of an individual's **behaviour** (Michie et al. 2011). **Capability** refers to the capacity of an individual to engage in an activity. **Motivation** is defined as conscious and sub‐conscious factors that influence behaviour and **opportunity,** as factors outside an individual that make a **behaviour** possible (Michie et al. 2011). The model has been used to explore SLTs’ views on helping parents of children with SSD to effectively deliver home practice (Pritchard et al. 2026), in early language intervention (Barnett et al. 2023) and in aphasia intervention (Johnson et al. 2021). Interventions for SSD, including home practice, have many factors involved and are therefore considered complex interventions. As such, it is vital to evaluate and consider the factors that drive the desired changes to behaviour through a theoretical framework (Skivington et al. 2021).

Adult learning theories in relation to effective coaching can also be applied to support SLTs to understand how they develop parental **capability** to ensure the efficacy of home practice (Pritchard et al. 2025). Effective parent training requires SLTs to have a thorough understanding of coaching strategies, underpinned by principles of adult learning theory (Leafe et al. 2025). Furthermore, Tosh et al. (2017) found that the successful implementation of home practice for speech and language intervention required explicit training for parents on how to complete the practice. Whilst many intervention studies for children with SSD include an expectation that parents will complete home practice (e.g., Flanagan and Ttofari Eecen 2018; Lim et al. 2020), the details of how parents are trained and what is expected of them is under‐reported in the literature (Sugden et al. 2016; Tosh et al. 2017). Only the most recent SSD intervention studies explicitly ground their approach to parent coaching within adult learning theory frameworks, providing specific details of the training and the expectations placed on the parents (Sugden et al. 2020). A review by Dunst and Trivette (2012) exploring what moderates the effectiveness of adult learning found that active involvement is key to knowledge and skill acquisition. Benefits to the learner were optimised when four or five different methods were used in combination. Providing opportunities for feedback and self‐reflection was important to learning (Dunst and Trivette 2012). SLTs play a key role in supporting parents to become involved in therapy sessions (Melvin et al. 2020). Since active involvement is key to effective learning, SLTs should be aware of the techniques that parents find the most effective to support their engagement in sessions.

It is also important to understand how parents perceive and experience relationships between themselves and the SLT, and between the SLT and their child. Current literature suggests that relationships are key to parents’ **motivation** for home practice for children with SSD (Leafe et al. 2025; Pritchard 2025; Sugden et al. 2019; Watts Pappas et al. 2016). Studies in the wider field of speech and language therapy report that developing effective relationships between clients and carers is key for engagement with and motivation for intervention (Connery et al. 2022; Melvin et al. 2020; Sylvestre et al. 2020). Developing effective therapeutic relationships is a crucial component in successful therapeutic alliances; these are essential for shared decision making and reaching the required intervention intensity, which in turn impacts intervention success (Sylvestre et al. 2020). Whilst existing literature highlights a lack of effective relationships leading to parents disengaging from SLT (Watts Pappas et al. 2016), little is known about which techniques are used by SLTs that parents perceive as effective for building these relationships.

Overall, there is limited literature exploring parental experiences of intervention and home practice for children with SSD. Two Australian studies have examined parents’ perceptions of their experiences with speech and language therapy for their child with SSD. One examined the evolution of a parent's involvement during a course of intervention (Watts Pappas et al. 2016), and the other explored parental experiences and preferences when completing home practice (Sugden et al. 2019). A Canadian study included parent interviews following delivery of a parent‐mediated intervention for children with SSD, looking at barriers and benefits to being involved in home practice (Lim et al. 2020). Examining strategies that SLTs use during intervention sessions, parents’ experiences of these strategies and their impact on home practice will inform how SLTs can work more effectively with parents. This will help SLTs determine what parents need from the SLT to ensure they feel sufficiently trained and confident to deliver effective home practice for children with SSD.

## 2 Aim

This study explores the experiences and perceptions of parents of children under 6‐years‐old with SSD who have engaged with speech and language therapy sessions and home practice. The focus is on how SLTs build successful relationships with parents and the techniques and strategies that parents have found successful in supporting them to complete home practice.

### 2.1 Research Questions

1. What is the experience of parents of children with SSD when working with SLTs in intervention sessions?
2. What do SLTs do in direct intervention sessions that parents perceive as working well to support them to deliver home practice?
3. What do parents think are the barriers and facilitators to carrying out home practice for their child with SSD?

### 2.2 Methodology

We took a critical realist approach, according to which reality exists independently of our perception, but our understanding of reality is shaped by experiences and interpretation is situated in society. This approach helped us recognise that participants' experiences were shaped by various factors, while also highlighting how our own knowledge and experiences influenced the data collection and analysis. Focus groups were initially chosen to allow for dynamic discussion, exploiting the interactive potential of the group. However, it was not possible to continue the study in this way due to participants’ conflicting schedules, and so we moved to 1:1 semi‐structured interviews. Like Lambert and Loiselle (2008), we found that the initial focus group findings allowed us to determine the most significant questions to explore further in the interviews, thus enriching the data we generated.

Ethical approval was obtained from University of Reading Research Ethics Commitee [2023‐223‐EP]. Data are reported using the Reflexive Thematic Analysis Reporting Guidelines (Braun and Clarke 2024).

### 2.3 Participants

Participants were parents of children under 6 years with SSD who had worked with an SLT within sessions and completed home practice. Data gathering was online via Microsoft Teams to increase the geographical reach of the study and accessibility of participation (Lobe et al. 2022).

### 2.4 Sampling and Recruitment

Participants were recruited from across the United Kingdom via email, professional networks, a university speech and language therapy clinic, advertising on social media and snowballing.

Sample size was guided by information power, considering the specific characteristics of the participants, and the narrow research aim as well as the team's experience in conducting qualitative research and expertise in the field of SSD (Malterud et al. 2016). We took an iterative approach to the data to determine its richness and discussed whether the data were sufficient to answer the research questions as we progressed, as recommended by Braun and Clarke (2021).

Twelve parents were recruited, and appointments offered included evenings and weekends. Despite this, only nine parents participated due to scheduling conflicts. Of the three parents unable to participate, two were fathers. Two parents were multilingual, but all participants spoke English at a level that enabled them to take part in the interview and in speech and language therapy sessions delivered in English. Participants had accessed speech and language therapy in a variety of sectors, geographical locations and physical settings (see Table 1). Four participants were parents of children in the Public and Patient Involvement and Engagement (PPIE) group (see PPIE section below for details). Some of the PPIE activity happened before the parent interviews, and so these parents may have had greater insight into the study than others.

**TABLE 1:** Participant characteristics.

Based on demographic information to indicate diversity of sample and potential barriers and facilitators to home practice as specified by others	*N* (total 9)
Parental role	
Mother	9
Child with speech sound disorder has a sibling	
Yes	9
Sector accessed SLT	
NHS	6
Private	4
University	5
Education	3
Multiple	8
Ethnicity (self‐reported based on office of national statistics classifications)	
White—English/Welsh/Scottish/Northern Irish/British	6
Mixed/Multiple ethnic groups—White and Asian	1
Mixed/Multiple ethnic groups—Any other mixed/multiple ethnic background	1
Other white background	1
Employment	
Full time	3
Part time	5
Not in paid employment	1
UK location	
Midlands	1
London and southeast	7
Northwest	1
Language/s spoken	
English only	7
English and Greek	1
English and Spanish	1
Participants known to first author prior to study	
Yes	4
Indices of multiple deprivation^a^	
7 9 10	2 3 4
Income Deprivation Affecting Children Index^a^	
4	1
7	3
8	2
10	3

^a^ Indices of multiple deprivation (IMD) and Income Deprivation Affecting Children Index (IDACI)—calculated through postcodes, where 1 = most deprived and 10 = least deprived https://imd‐by‐postcode.opendatacommunities.org/imd/2019 (calculated—10 March 2026).

**FIGURE 1:**
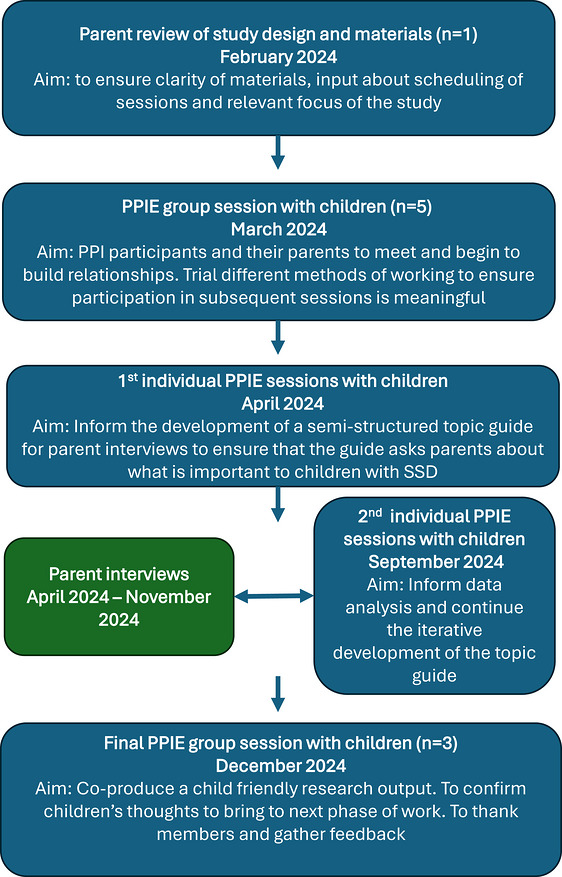
Interaction between PPIE and interviews

Figure 1 demonstrates the interaction between the PPIE sessions and the parent focus groups and interviews.

### 2.5 Data Generation

Three parents participated in a 2‐h focus group. This was followed by 1‐h individual interviews with six further parents. Focus groups and interviews were all facilitated by either KP, EP or JT and took place between April and November 2024. Where KP had an existing relationship with a parent, the sessions were led by EP and/or JT without KP present to avoid any potential bias of response.

**FIGURE 2:**
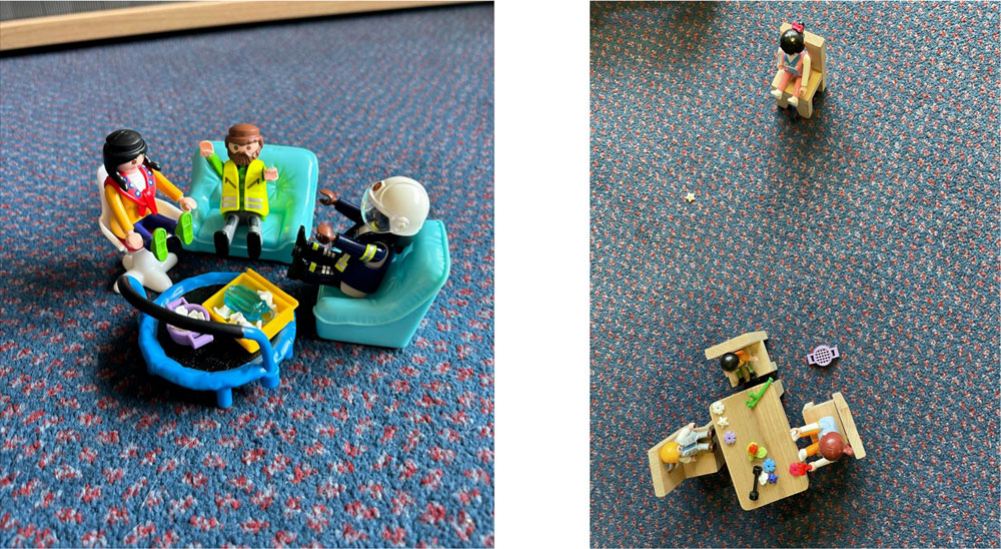
Example slide

Sessions followed a semi‐structured topic guide, which was used iteratively. The topic guide addressed the research questions and drew on previous research by the team (Pritchard et al. 2025, 2026), adult learning theory and the COM‐B model (Michie et al. 2011). Children's quotes, photographs and drawings from the PPIE sessions were used to trigger discussions throughout (see Figure 2 for an example). At the end of each section of the focus group/interview, the researcher summarised the key points and invited the parents to comment, expand or clarify any of the points they made, with a further opportunity for this at the end of the session. An email was sent following the session, giving the parent a further opportunity to comment on their contributions. Focus groups and PPIE sessions informed the development and refinement of the guide for the subsequent interviews. See Appendix 1 for the topic guide, including details of how PPIE, COM‐B, adult learning theory and previous research impacted the design.

Sessions were video‐recorded via MS Teams. Automatic transcripts were checked by KP for accuracy and transcribed verbatim with identifying details removed.

### 2.6 First Author's Reflexivity

Throughout the study, we took a reflexive approach with KP keeping a reflexive diary and regular discussion with the whole team. Appendix 2 details KP's reflexivity and positionality, including how KP's clinical experience and assumptions influenced the analysis.

### 2.7 Analysis

During our initial analysis, we took a predominantly inductive approach to allow for novel concepts to be generated, as appropriate for qualitative studies focusing on human experiences (Bradbury‐Jones et al. 2017). This allowed us to become familiar with the data and ensure we understood the participants’ intended meaning. Following this, we took a mixed inductive/deductive approach, recognising that existing knowledge had informed the data generation and thus the analysis, whilst still being open to new ideas (Braun and Clarke 2021). The team's knowledge of the SSD literature and theoretical frameworks, such as behaviour change (Michie et al. 2011) and adult learning theories such as Dunst and Trivette (2012), was considered. During the later stages of analysis, we used the COM‐B model (Michie et al. 2011) specifically to frame our results, examining parental perspectives on how SLTs had supported parents to develop their **capability** and **motivation,** as well as ensure **opportunities** to develop the desired **behaviour** for home practice. We used reflexive thematic analysis (TA) (Braun and Clarke 2021). Reflexive TA aims to interpret patterns of shared meaning from the data and emphasises the researcher's role in this analysis. We chose a reflexive TA as KP is a clinically active SLT who works with children with SSD and has a specialist interest in SSD. It was therefore important to take a reflexive approach to analysis to ensure we were mindful of how experiences shaped the study. We used the six stages of reflexive TA to guide our analysis as follows:

1. Familiarisation—starting during data generation and continuing through all stages of analysis as the transcripts were read and reread.
2. Coding—assigning codes to data, providing analytical descriptions. Being open to novel ideas whilst also considering the data in relation to previous research and theory. We came back to this phase several times as we generated themes to make sure that the codes and related data extracts captured single units of meaning. We developed semantic and latent codes with no prioritisation of one over the other to capture both the surface meaning and that which was implied by the participants (see Appendix 3 for examples). At this stage, we began considering the data in relation to the previous research, for example, consistent with (Pritchard et al. 2026), we noted that each parent mentioned multiple strategies used to support their **capability**, as is recommended in adult learning theories (Dunst and Trivette 2012).
3. Generating candidate themes—organising codes into groups of shared meaning. At this stage, we worked with the PPIE group as detailed in the PPIE section below and in Appendix 4.
4. Developing and reviewing themes—this was an iterative process and involved KP writing summaries of the candidate themes to share with the team for comment and discussion. At this stage, we went back to the raw data to ensure that the themes captured the meaning and began to map the themes to the COM‐B model (Michie et al. 2011).
5. Refining, defining and naming themes—our discussions at this stage were to ensure that each theme was clearly separate and the names captured the core shared meaning. Our aim was for the theme names to go beyond topic labels and capture a ‘central organising concept’ (Braun and Clarke 2006, 35).
6. Writing up—our analysis continued throughout the writing up, going back and forth through the previous stages to ensure the outcome of the analysis made sense in relation to the data (Braun and Clarke 2021), the research questions and the COM‐B model.

Whilst these stages are presented in order, the analytical approach was not linear and rather went back and forth fluidly between the data, codes and themes.

All analysis was completed by KP and discussed with the rest of the team to refine thinking and sense check the narrative of the analysis. As per Reflexive TA, the aim of these discussions was not to come to a consensus but to share ideas, consider our own impact on the interpretation and ensure that KP was able to explain her interpretation clearly and support it with relevant data (Braun and Clarke 2021). Analysis finished at the end of phase 6 when we were happy that the research questions had been answered, the themes were separate from each other, their names captured the shared meaning, and that our analysis made sense in relation to the COM‐B model.

### 2.8 Public and Patient Involvement and Engagement (PPIE)

A parent of a child with SSD provided feedback on the study design and materials. The materials were shared via email, and a list of questions was provided for guidance, as well as opportunities to add additional information. The aim of this review was to

- ensure the participant information sheet was clear
- ensure the topic guide and aims of the study focused on areas relevant to parents
- gather thoughts on timings and emphasis of the planned sessions.

In addition, five children with lived experience of SSD, direct intervention targeting their speech and home practice, formed a PPIE group. The children were identified as having a diagnosis of SSD by their SLT. All children were White British and were monolingual English speakers. Three children had attended speech and language therapy delivered by the lead author. Therefore, activities asking directly about experiences of speech therapy sessions for these children were facilitated by the supporting researcher in the session to reduce potential bias (see Table 2 for more details). The children attended in person with their parents. Parents supported their child's communication as required but were not members of the PPIE group. An initial group session trialled ways of working, such as drawing, use of a visual timetable, rating scales and ranking activities, to determine what methods would best enable successful communication for each child. The subsequent sessions were then designed using the most successful methods for each child to ensure meaningful and impactful contributions. The children supported the study as follows:

- Developing and refining the topic guide—for example, children were asked to draw a picture of themselves completing home practice—ideas from these drawings were used to produce questions and prompts. The drawings were then used to elicit conversation with parents during the interviews.
- Informing the results during the early stages of analysis—for example, statements relating to the codes and candidate themes were presented to the children, such as, ‘I can choose when I want to practice’, relating to the code ‘practice sessions are guided/led by child's choice which impacts engagement’. Children were asked to indicate their agreement in terms of ‘yes, no’, don't know’. This information helped to shape our thinking around the suitability of codes, how they related to the themes and the impact the final results would have on the children who experience intervention for SSD.
- Creating a child‐friendly research output video to highlight the impact of PPIE and allow the outcomes to be shared with an audience outside of academia, as recommended in UK standards for public involvement (NIHR 2019).

**TABLE 2:** Public and patient involvement and engagement group

Pseudonym—chosen by child	Age (years) at start of study	Gender	Age in years when first known to SLT	Sector where SLT is accessed	Receiving SLT intervention at the start of study	Pre‐existing relationship with the lead author	Child's parent participated in an interview/focus group
Mario	5	Male	4	University	Yes	None	No
Leo	6	Male	4	University	Yes	SLT	Yes
Harlequin	5	Female	3	University and private	Yes	None	Yes
Foxtrot	5	Male	2	NHS and university	No	SLT	Yes
Ruku	4	Female	3	NHS and university	Yes	SLT	Yes

Further examples of the methods used and the impact on the research can be found in Appendix 4.

## 3 Results

**FIGURE 3:**
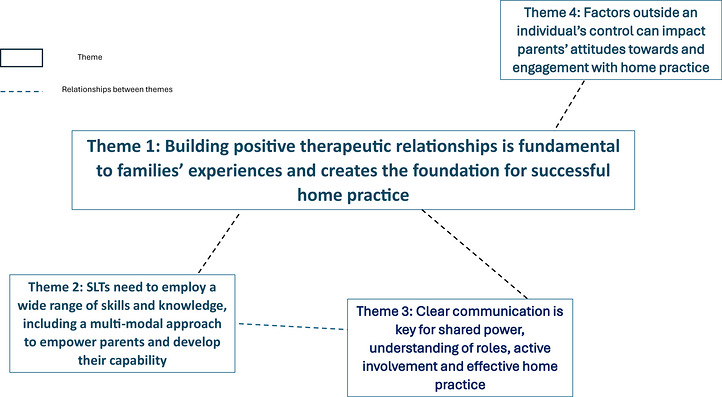
Diagram of themes

**FIGURE 4:**
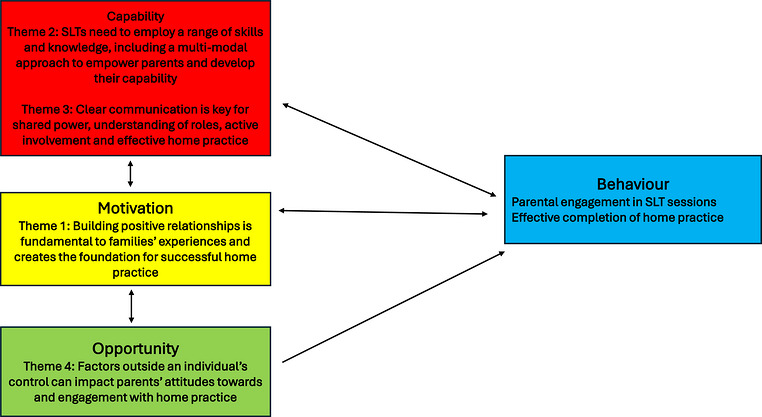
Themes within the COM‐B model

We explored the perspectives of parents of young children with SSD on their experiences of intervention and home practice for their child's speech. We were specifically interested in how SLTs build relationships with parents and what they do within direct therapy sessions to enable parents to become successful implementors of intervention at home. We constructed four themes (Figure 3) and examined them in relation to the COM‐B model of behaviour change (Michie et al. 2011) (Figure 4). This allowed us to explain what parents need to develop their **capability**, **motivation** and to provide appropriate **opportunities** to ensure the desired **behaviour**—engagement within intervention sessions and home practice delivered accurately and at the appropriate intensity.

### 3.1 Theme 1: Building Positive Therapeutic Relationships Is Fundamental to Families’ Experiences and Creates the Foundation for Successful Home Practice

Our findings indicate that relationships and the skills to build a relationship with a child and their parent play a central role in successful speech and language therapy and the **motivation** to complete home practice. This links with studies which have found that SLTs forming an effective relationship with clients and/or their carers leads to a therapeutic alliance between the clinician and the client(s) which fosters patient‐centred care and successful outcomes (Sylvestre et al. 2020).

Our parents often had experiences with several different SLTs and reflected that the difference between the SLTs who provided effective intervention sessions and those who were less effective was the SLT's skills at engaging with and building a relationship with their child:

*‘Our original speech therapist went on maternity leave and we hired someone else in the interim, […]* 1 *and it just wasn't a good fit. My child needed someone […] it was really important that the speech therapist brought a lot of energy to the session. […], And the last thing we wanted, I think there was she's only ever had one session where she went, do I have to go to talking today? and, […] that worried me. 'cause I don't want her to ever feel like it's a massive chore.’ (P5)*

Later, they go on to say the following:

‘The speech therapist that we left, she had the same organisation. She had the same games. Everything was almost exactly the same. […] there was essentially the same components there to each session. I would say the first thing would be energy and excitement for the child specifically.’ (P5)

P5 reflects on the fact that each SLT did the same thing, but the difference between them was the ability to engage and build a relationship with their child. Part of the skill in building that relationship was the SLT's ability to make the sessions exciting. In this case, the lack of relationship led the child to become disengaged with speech and language therapy and ultimately led to the parents withdrawing the child from that SLT. The **motivating** factor here is the relationship between her child and the SLT.

As well as impacting the child's motivation, the relationship also affects the parent's role within the sessions themselves. P3 has experience with multiple SLTs and felt able to compare:

*‘I think probably the ones who were, […] the same person who was, who I thought was lovely with him. I then liked automatically and then I felt comfortable with. […] the ones who weren't quite as warm and friendly with x and I, I felt like I was having to work hard to be nice and enthusiastic in the session.’ (P3)*

P3 recognises that some SLTs were effective at forming a therapeutic relationship with them and some were less so. This then changed their role within the session as they felt the need to support their child's engagement. This potentially had an impact on the parents’ ability to learn from the session and thus develop their **capability** for home practice. This parent also highlights that the impact of building an effective relationship with her child led to her feeling more positive towards the SLT. Previous research has highlighted how all the relationships within the speech and language therapy process are intertwined and thus impact each other (Pritchard et al. 2025).

**FIGURE 5:**
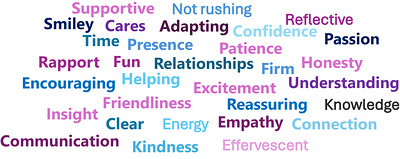
Word cloud—‘what are the important qualities of an effective SLT?’

The importance of the relationship to a parent's experiences with speech and language therapy was highlighted when participants were asked what makes an effective SLT. Figure 5 presents the words that parents used in response to this question, highlighting that qualities that support a good relationship are the most valued.

Other parents reflected on different aspects of the SLTs’ ability to support them and their child emotionally.

*‘I think kindness comes and patience and just the tone of voice. Everything's very nice. […], everyone's super super nice and kind and smiley and calm and patient, so it feels it's a it's a nice feeling. That's what I feel. I'm sure that's what she [the child] feels and in the [SLT] students as well. […] everything's lovely and and everyone's got ooodles of patience. Which um, I thought was amazing that [SLTs] could like sort of show that to her.’ (P9)*

The parent reflects on the importance of these qualities to her child. During the interview, this parent discussed how hard her child could find sessions due to possible attention deficit hyperactivity disorder, the SLT's acceptance of the child and the value of the unconditional support that the SLT demonstrated was valued.

A positive therapeutic relationship with the SLT can enhance a parent's **motivation** to engage in and complete home practice. Later, whilst reflecting on the positive experiences that they and their child have with their SLT, P9 responded to: ‘did you think it helped having that good relationship?’

*‘Yeah. Yes. Yeah. Yeah. Because I didn't want to let [SLT] down as well because you think they've given all this time to us, […] not only do we need to do it, because we need to do it, […] I didn't want to come back and say we hadn't done everything, you know, because, […] it's, you know, it's respectful, isn't it too. You give us time, we we took it, take it seriously.’ (P9)*

The emphatic *‘yeah…’* highlights the importance of the relationship to the parent. The SLT taking the time to work with them and build those relationships **motivates** them to complete the home practice. There is a sense of accountability from the parent that this relationship brings, as well as the parent recognising the importance of the home practice itself.

Conversely, when a relationship is not going well, this can be de**motivating**, leading to a parent not completing the home practice, acting as a barrier to the desired **behaviour**:

*‘I was quite stressed because sometimes they were guiding me on. ‘Ohh you need to push them or you need to give them a reward’ which this doesn't resonate with my parenting style and my theory of of parenting. So for me it was very hard to impose this practice, when I disagree with it basically, […] so for me that was very hard as well to to try and impose something that I totally disagree with.’ (P2)*

There is a sense of struggle here, implying that the parent feels like the SLT is working against them. It is possible that if the SLT had explored the parent's ‘*theory of parenting’*, then they could have adapted the approach taken during the therapy sessions and home practice to support the parent's **motivation**. Sugden et al. (2019) also found that one reason parents did not complete home practice was due to the activities not being suitable for the child or family. This highlights the importance of understanding the parent's context and beliefs and not making assumptions about what they feel comfortable with.

### 3.2 Theme 2: SLTs Need to Employ a Wide Range of Skills and Knowledge Including a Multi‐Modal Approach to Empower Parents and Develop Their Capability

During the construction of this theme, we explored the range of strategies and skills experienced by our participants and how this developed their **capability** to deliver home practice successfully. Many parents acknowledged that their understanding of SSD was important, and when SLTs demonstrated knowledge and took time to explain SSD, it fostered a sense of trust in the therapist. However, it was widely acknowledged that knowledge was not the only thing needed to be a successful SLT. A much more varied skill set was required.

*When Asked ‘What Makes an Effective SLT?’*

P6 responded the following:

*‘Fundamentally I think […] you have to be able to have a good rapport with children and the way you communicate with children has to be, especially young children, it has to be fun, you know, so fun, I think, reflective and reflective I think is really important. So getting to know the child, thinking about what's gone well, what hasn't and then adapting it for the follow up session as opposed to just kind of doing the same, if you're not getting the right outcome, don't carry on doing the same approach. […]*

*If you've got the knowledge but you're unable to put the knowledge into practise effectively, then it's not really much, not really much use.’ (P6)*

This quote is in the context of P6 having what they considered to be a negative experience with their child's SLT. They may be reflecting on what was missing for them. The SLT, having more than just knowledge, would have both supported the building of effective relationships, as well as supported intervention outcomes using self‐reflection and flexible delivery, adapted for the individual.

Being actively involved in the session was seen to be supportive in building parental capability, and parents reported that even when they were observing, having something active to do, like a checklist, supported them to feel engaged in the session and enabled them to know what to look out for at home. Here, P4 describes their SLT sessions:

*‘I guess it's like showing the parent and then it would be getting us to actually then implement the method ourselves. And I think that worked really well. It's it's the best way of learning for anyone and everyone is to is to get involved and you do it yourself, […] to get the pointers actually from the professional as to what you might want to try. […] And I thought that was really useful, really useful way of teaching us and equipping us as the parents with the tools that we needed to um then do it at home.’ (P4)*

They reflect on a variety of strategies that supported them to feel **capable** at home. The combination of observation, discussion, active involvement, feedback and self‐reflection in this example allowed the parents to feel **capable** of completing home practice. Highlighted here is the importance of doing the home practice activities in the session with the SLT there to provide feedback.

The parents also emphasised that the approach should be delivered through play. An SLT's skill in making sessions fun was felt to be essential to supporting both their child to engage in the sessions and to support home practice. Without this, parents felt that their child would not have engaged in the sessions, making it harder for the parent to motivate their child to complete home practice:

*‘If it was more if it was like structured and you had to sit down. You have to do this and repeat, repeat, repeat and not have games. I don't think it. I don't, he wouldn't enjoy it and then he wouldn't do it.’ (P8)*

P8 recognises the need for repetition in the practice and the important role that games play in motivating their child to complete the practice. Other parents reflected that practice had to feel different from schoolwork so that their child did not perceive home practice as additional work. Differentiating school and speech work has been found to be important in other studies of parents’ experiences (Watts Pappas et al. 2016). Making sessions fun in this way requires SLTs to be creative to ensure that each child they work with engages with the sessions. This creativity was valued:

*‘My dealings with the therapist is just, yeah, willing to go above and beyond. And I think sometimes think outside the box as well. Which is important because and as I've as I've said even before, like it's not a one‐size‐fits‐all with, particularly preschoolers […] being able to think, OK, well, that technique didn't work with that child, right! Let's have a think.’ (P4)*

Like P6, P4 draws on the importance of the SLT's ability to reflect on their practice. What they highlight is that this ability to reflect and think creatively also allows the SLT to think flexibly, to individualise the intervention. This flexible thinking is another valuable skill supporting the therapeutic process. The need for flexibility and individualisation has been discussed in previous studies as important for supporting parents (Leafe et al. 2025; Pritchard et al. 2025). This finding emphasises how SLTs working flexibly and adapting to the individual child and family support successful home practice.

Table 3 summarises the strategies that parents discussed that helped them to feel capable of delivering home practice effectively.

**TABLE 3:** Strategies that support parental capability and home practice.

Strategy or technique	Supporting quote
Observation of SLT working with child	‘I'm really keen to learn and to do, to do it, but I'm gonna need you to show me.’ (P4)
SLT providing checklists for parents to complete during observations.	‘When I've been a bit further out, I will have that [a checklist] in front of me with a pen and I'm ticking. And then [SLT] come over every now and again and ‘Ohh he's done his ticks’’ (P1)
Providing clear instructions	‘Give me clear instructions each week about what we need to concentrate on and what not to.’ (P9)
Parent taking notes	‘I take notes during the session or I'll put notes on my phone.’ (P8)
Parent trying out therapy in session	‘getting us to actually then implement the method ourselves. And I think that worked really well.’ (P4)
Supporting the parent to understand the child's diagnosis/child's perspective	‘What we, I found most valuable as well is is just understanding my child's condition. […] I've had explained to me at speech therapy […] just understanding what it's like to live in my child's world. That, as a parent, is invaluable because I didn't have that insight before.’ (P5)
Supporting the child to understand the rationale for speech and language therapy	‘He understands ultimately why he's there. But as it happens, he tends to do really well because he wants he wants to do it.’ (P1)
Use of technology and making practice different from school work	‘The introduction of the iPad because it's different to what he does with school work. School work's all books and writing, and I think the flash cards again, he was just like oh really? […] But yeah, with the iPad it's something a little bit different’ (P7)
Instructions on how to use the technology	‘we're not very good with technology. So we were trying, […] trying to access the app and download it. […] I mean, I'm sure if they'd set it up for us on her iPad, her tablet, we would have had a bit more success, but it was just that kind of go home download this.’ (P5)
Flexible and individualised approach to therapy	‘Think outside the box as well. Which is important because […] it's not a one‐size‐fits‐all with, particularly preschoolers. […] being able to think, OK, well, that technique didn't work with that child, right! Let's have a think.’ (P4)
Providing fun resources	‘They gave us games to do again, I'm not, I'm not creative at all […] So I needed help with that.’ (P3)
Providing feedback to the parent	‘The therapist sort of giving a bit of feedback to then us as the parent’ (P4)
Opportunities for parents to self‐reflect on home practice	‘To say ah, ‘like how how was that for you?’ And that was always a good thing, actually […] that's that's good to actually ask the parent like are you, you know, how do you feel about it?’’ (P4)
Using videos of sessions	‘letting me agree to let me film her [SLT]. That helps, because I find I stopped her in sessions and I'll go, ‘No, let me record this,’ because it just helps’ (P8)
Providing parent‐only training sessions	‘I do think, it might be beneficial to have like a parent only consultation. […] Yeah, talk about it without maybe the child present.’ (P7)
Facilitating peer support for parents	‘It might be nice actually to […] have some sort of like parental group chat where parents can support each other that with like with children that have similar sort of speech.’ (P7)
Recaps to monitor home practice	‘We would have a little chat at the beginning sort of find find out how the last week's almost like practise at home had gone, whether those methods had been effective, did they work well, did they not work so well.’ (P4)
Guidance on what to do if a child is struggling at home	‘at the beginning I was like bit very unsure doing it with him at home because I didn't want to get wrong and then but then like the SLT always says, like if he's starting to repeat things incorrectly, then we just stop, let her know for the next session and then we can move on to the next word but before I didn't know what we had to do really.’ (P8)
Room set up that invites a parent to join in	‘it's [the room] set up with the option of me to sit at the side, but enough chairs that I can sit and be part of the session as well. So I feel like I've got the option, […] very quickly, you realise she needs that support […] it would be really strange for me just to be there, but not taking part, She'd, I think, she would feel that that it's strange as well.’ (P9)
Use of visual support, such as diagrams	‘She used to like to draw like diagrams, and then how it works or not from the ear to the brain…So that that helps’ (P8)

### 3.3 Theme 3: Clear Communication Is Key for Shared Power, Understanding of Roles, Active Involvement and Effective Home Practice

Parents perceived the clarity of the SLT's communication as impacting their confidence within the sessions and their **capability** at home. This theme is closely linked with both Themes 1 and 2. Clear communication is important to develop successful relationships, which supports **motivation** (Theme 1), and many of the skills and strategies employed by SLTs to develop parental **capability** also require clear communication (Theme 2). Parents who reported a negative experience within the SLT sessions often spoke of not knowing what to do. This lack of clarity impacted their involvement in the session, as seen in P3's comments:

*‘I tried to stay quite quiet […] I didn't have much guidance of what I should have been doing. She didn't say at the start of the session. Right, I'm gonna do these things with x, […] You know, there was, there was no guidance to me at all.’*

*[OK. And would that, have been helpful?]*

*‘Definitely, definitely [nods emphatically].’ (P3)*

The impact of the lack of guidance on the parent in this example is that they became a passive observer within the session. They acknowledge that they would have been happy to be involved in the session, but the lack of clarity about their role meant that they did not feel comfortable getting involved. This led to a power imbalance between them and the SLT. This has been found in another study where parents of children with SSD were concerned about interfering with the speech and language therapy sessions (Watts Pappas et al. 2016). Other parents we spoke to indicated that the SLT explicitly invited them into the session, and this resulted in their active participation; these parents also indicated that without this explicit invitation, they would not have become involved. Where SLTs provided clarity, this was done through verbal instructions and in how the room was set up, for example, having enough chairs around the table for the parent. Understanding of parent/SLT roles is key to satisfaction and trust with an SLT (Leafe et al. 2025). SLTs can use their clear communication skills to consider how they break down any power imbalances to allow for parental involvement, which may in turn support a parent's **capability** within the session and beyond.

Other parents spoke about the impact that the lack of clear guidance had on their ability to complete the home practice:

*‘There was nothing. There was no kind of like. It was just kind of like you need to practise. And practise was just the sort of you need to “practise.” [makes quotes gesture], you know, “practise” and then that was it. But I always used to sort of go thinking of course I'm going to be asked if I've “practised” and we haven't “practised” much.’ (P6)*

P6's comments indicate that what they required was clarity about what they should have been doing at home. The participant feels accountable for the practice but does not understand what is expected of them, due to a lack of specificity from the SLT, and this feels hard.

Clear communication is also needed when supporting parents to make sense of terminology, diagnosis and intervention approaches (e.g., McAllister et al. 2011). For our parents, understanding supported their **motivation** to practice at home. The parents reported feeling confused by terminology and needing to look things up following a session or after a report had been written. Those who felt clear in their understanding discussed ways in which SLTs had supported them to unpick complex theories and concepts about speech production. Such support is discussed by P8:

*‘She used to like to draw like diagrams, and then how it works or not from the ear to the brain…So that that helps 'cause, it's like, because sometimes you think, why isn't he getting it? […] Then she just she draws diagrams about how sometimes when it goes in then it comes out differently when it comes out…Because sometimes you feel 'cause like we're not trained, we don't know. And it's like you hear that sound and then it's like, but why can't you say it? Yeah. So that that helps because you've then got more understanding.’ (P8)*

Here, the SLT used drawing to support the verbal explanation to ensure that the parent understood what was going on for their child. The parents’ explanation of the approach indicates how they find this multi‐modal approach to communication clear and feel confident in their understanding of their child's SSD. Converting complex ideas and using the full range of verbal and non‐verbal communication skills may therefore be required to support parents to understand SSD, thus **motivating** them to continue at home.

### 3.4 Theme 4: Factors Outside of an Individual's Control Can Impact Parents’ Attitudes Towards and Engagement With Home Practice

This theme explores the different barriers and facilitators experienced by parents that impact the **opportunities** for home practice and for developing their **capability**. These factors can be outside of the parental and the individual SLT's control. These factors not only influence the **opportunities** available but can also impact parents’ views, experiences of and **motivation** for home practice.

Most of the parents we spoke to had experience of more than one SLT, across multiple sectors (Table 1). Often, their experiences with their SLT or the model of service delivery did not meet their expectations. This was also seen by Watts Pappas et al. (2016) in their interviews with parents. Here, P1 talks about their initial experiences with speech and language therapy:

*‘I think as long as you can show the parent and you are seeing them regularly, you know weekly and then they go off and do it that week, that's fine. But in my case, at one time, you know, we weren't being seen every three, six months, to leave me to go home to do that speech therapy just wasn't, in my opinion, OK. … we we've gotta get it really right that parents are involved and are doing that at home. It's that fine line and making sure that the the that they're there to help and to reach out to, because like I said in the particularly the earlier days at it was every six months and then I, I'd be sort of sat there having to show them what, I'd been doing and yeah, it it didn't always feel that we were working together.’ (P1)*

P1's initial experiences with SLT did not meet their expectations. The long gaps between sessions and the lack of contact with the SLT in between sessions led to them not being clear about what to do. The implication from the extract is that the gaps between appointments led to a lack of partnership between them and the SLT, leaving them feeling unsupported. This demonstrates how the model of service delivery can create a barrier to effective home practice, especially when there are long gaps between sessions. Regular contact between the parent and the SLT is important for a parent to feel supported. For some parents, the limited service and the disparity between expectations and reality led them to feel desperate, having to fight for the support. This led to one of the parents moving to her native country, with her child, away from her husband and son, to receive 3 months of SLT. Service delivery factors and dissatisfaction of service models have also been identified as important factors impacting intervention in other studies examining parents’ experiences for children with SSD (Watts Pappas et al. 2016).

Like P1, P2 experienced gaps between appointments, which led to her struggling with home practice.

*‘I had to remember to remodel this and play with my child for the next 6 to 8 weeks when the next visit was due, more or less, it, sometimes it was even longer. […] I felt the extreme extreme pressure to be able to find time to do these activities.’ (P2)*

The lack of contact with the SLT between sessions has led to the parent feeling stressed, and there is a sense that the whole responsibility for their child's progress is with them. Not only is the lack of regular contact potentially having an impact on this parent's well‐being, but it also raises concerns around models of service delivery restricting the SLT's ability to deliver evidence‐based intervention intensity (Allen 2013; McFaul et al. 2022). Whilst we did not collect information about the severity of SSD or about the exact models of service delivery, what the parents reported would not have allowed for the effective frequency of sessions. Most parents we spoke to recognised that individual SLTs were not to blame and were delivering the service in the way they had been directed.

The way in which a service is delivered not only impacts the parents’ confidence and **capability** with home practice but can also impact the trust in the process and in the SLT, and this can then impact the relationship. P4, who has had experiences with four different SLTs, across independent and NHS services, reflected on this:

*‘We had been seeing the therapist at the clinic for a little while. I I think I felt level of trust, in them as well to, to be honest, that and I think that's important rather than perhaps you know a sort of slightly different relationship. Seeing a different therapist for, you know, one off, maybe over three months and yeah that, that that route didn't work in in our case anyway.’ (P4)*

P4 recognises that building relationships takes time, which isn't always accounted for in the way the service is delivered. This indicates that it may be important for services and individual SLTs to allow time for relationship building and developing trust to ensure that the parent is on board.

Studies show parents often struggle to find **opportunities** for home practice in their daily lives and may not always complete it as assigned (Sugden et al. 2019; Watts Pappas et al. 2016). Within our participants, some parents found fitting the practice manageable, while others, like P9, found competing demands made it difficult:

*‘I've got two other children and just the general doing daily things […] It was more about just fitting it in and just taking time to. […]. It's just not having enough time in the day I think to fit it in. […] I know it shouldn't be an excuse, but that's probably why it didn't happen every day.’ (P9)*

They perceive daily life to be a barrier to home practice, demonstrating that other pressures in a family's life can conflict with home practice. The reference to ‘an excuse’ suggests that there are feelings of guilt around not being able to complete the home practice as recommended.

Parents highlighted the importance of developing the **capability** of more than one person where possible, noting challenges when only one person could attend. Difficulty relaying information to the other parent, added pressure and reduced effectiveness of home practice. Families with two parents able to attend, like P4, saw clear benefits:

*‘it's important that, where possible, that both parents are like trained up as it as it were, if that makes sense? […] So, it's getting dad to feel confident with what he's doing as well. […] because I think children can respond differently as well.’ (P4)*

P4's comment not only emphasises the value of additional people being involved but also demonstrates the role of the different relationships that children have with different adults and the impact of these on home practice. An SLT may have to individualise their support to each person, even when working with the same child, which may be difficult if the SLT only meets one parent. Other parents spoke about how their parenting partner completed home practice but was unable to attend the sessions with the SLT due to work commitments. The reality of only one parent being able to attend therefore has the potential to reduce **opportunities** for and the **capabilities** of the non‐attending parent or other key adult to support home practice effectively.

All the parents involved in our study had more than one child, with the number of siblings ranging from one to three. There was a conflicting opinion as to whether siblings created a barrier or a facilitator to home practice. Some parents were clear that having multiple children could make completing home practice more difficult. However, other parents spoke about creating **opportunities** by getting the whole family involved, giving the child a set number of words to practice with each family member, or asking siblings to create speech games to play. Those whose children had older siblings were more positive about the impact of siblings than those who had younger children. P8 was particularly enthusiastic about the role her older child had in facilitating the practice. He had attended intervention sessions in the holidays during which he received guidance from the SLT about how to support his brother:

*‘We get his brother involved as well, because then he works better with his brother for some reason.’ (P8)*

Here, siblings play a role in home practice, creating additional **opportunities** for home practice. P8 reported that their child's brother enjoyed attending SLT sessions, and had a positive sibling relationship, plus developing the brother's **capability,** through attending speech and language therapy sessions with the SLT, appeared to support home practice.

## 4 Discussion

Our study set out to explore parental experiences and perceptions of working alongside an SLT within intervention sessions and at home to support their child with their speech and language therapy for SSD. Our focus was on the elements of speech and language therapy sessions that parents felt enabled or prevented them from delivering effective home practice with their child for speech.

Our study was guided by the COM‐B model of behaviour change (Michie et al. 2011), which enabled us to consider factors that may drive the desired behaviour of parental engagement with intervention sessions and home practice. We were interested in how SLTs developed parents’ **capability**, how they maximised **opportunities** and how they supported parents’ **motivation** for home practice with their children with SSD. Using this model allowed us to identify barriers and facilitators that contribute to a parent's engagement with intervention sessions and home practice.

### 4.1 Capability

Key to developing parental **capability** for home practice for children with SSD is the utilisation of a combination of different approaches (Leafe et al. 2025), aligned with adult learning theory, where adults learn new skills and knowledge best when four or five different methods of coaching (such as observation, written information, self‐reflection) are combined, with active involvement being key to knowledge and skill acquisition in adult learners (Dunst and Trivette 2012). Our participants spoke of a wide range of strategies that supported them to feel confident in their ability to deliver home practice effectively, and multiple strategies were often discussed by the same parent, indicating that a varied approach to the coaching and support of parents was implemented by SLTs to ensure their **capability**.

Previous studies have indicated that parental understanding of their role as implementors of intervention can change over the time they are involved with the intervention (Davies et al. 2017; Tosh et al. 2017; Watts Pappas et al. 2016). Where our participants felt secure in their role as implementors of intervention, clear communication and the use of a variety of means of communication (written, diagram, videos, observation) were discussed as important. This communication supported parents’ understanding of their expectations of roles and the rationale for the methods adopted by the SLT. The need for clear communication to support parents to understand their role and the SLT's rationale has emerged in other studies looking at home practice for speech (e.g., Sugden et al. 2019).

Parents who reported that a positive experience with home practice felt that active involvement within the sessions, including feedback on and reflection of their ability, was key to their success. Theories of adult learning suggest that active involvement and self‐reflection are key to enabling adults to learn (Dunst and Trivette 2012). Additionally, active involvement within sessions has been found to be key to engaging parents within the wider field of SLT (Melvin et al. 2020; Melvin et al. 2023). Our findings suggest that this also applies to parents of children with SSD.

Despite many of our participants feeling confident in their role as implementors of the intervention, many parents felt they did not have the **capability** to complete home tasks effectively and, as a result, did not always complete the practice. Parents are more likely to complete home practice if they believe they will be successful (Leafe et al. 2025). Moreover, the Health and Care Professions Council's standards of conduct, performance and ethics states that SLTs must ‘only delegate work to someone who has the knowledge, skills and experience needed to carry it out safely and effectively’. (standard 4.1, HCPC, 2024). Training and upskilling parents effectively is thus important to ensure SLTs are adhering to professional standards.

### 4.2 Opportunity

Our findings indicate that barriers to **opportunities** for home practice are mostly outside of parents’ or SLT's control. Some of the factors discussed by our participants, such as having siblings, could either be a barrier or a facilitator depending on the specific family context and age of the sibling/s. Including siblings to maximise **opportunities** for home practice is a novel finding. In previous studies, parents of children with SSD have discussed siblings in the context of managing the practicalities of daily life, thus perceiving siblings as a potential barrier to overcome rather than a possible facilitator (Sugden et al. 2019). Our study suggests that SLTs actively involving siblings in home practice could be potentially beneficial for some families. Whilst there is evidence that intervention for SSD can be effective when supported by a trained adult, such as a parent, completing home practice (Scherer et al. 2008; Sugden et al. 2020), direct intervention with an SLT is also required for a range of SSD diagnoses (McCabe et al. 2024; RCSLT, 2024; Sugden et al. 2018). Parents in our study reported long gaps between direct SLT‐led sessions and felt that these gaps reduced their **capability** and **opportunities** for effective home practice due to limited face‐to‐face time with the SLT. The reduced frequency of sessions also has implications for the overall outcome of speech and language therapy as intervention for SSD has been found to be more effective when delivered at a higher frequency, when compared with lower session frequency. For example, Allen (2013) found that the same number of sessions was more effective when delivered three times a week over 8 weeks, than once a week for 24 weeks. This gap between what is reported in the literature and what happens in clinical practice experienced by our participants has also been reported previously (Hegarty et al. 2018; Oliveira et al. 2015; Sugden et al. 2018). Moreover, intervention context, including service constraints, such as a limited number of appointments, is the primary external factor negatively affecting the therapeutic alliance (Sylvestre et al. 2020). Offering the same number of appointments over a shorter timeframe could support SLTs to deliver a service that is more aligned with the evidence base (McFaul et al. 2022). In addition to supporting evidence‐based session frequency, our study suggests that offering the same sessions over a more condensed period could also provide more **opportunities** for building relationships and training parents to allow them to become skilled implementors of intervention at home and thus maximise the efficiency and effectiveness of the service.

### 4.3 Motivation

Despite varied SLT experiences, the child–SLT–parent relationship was the factor that most shaped our participants’ perception of intervention sessions. All the participants agreed that an effective therapeutic relationship was essential to ensure a positive experience of working with the SLT in the therapy sessions. Like in previous studies (Leafe et al. 2025; Pritchard et al. 2025, 2026), relationships appear to be a key **motivating** factor for parents. For some of our parents, developing an effective relationship was the difference between a parent engaging with the SLT or not. Parents disengaging with SLT intervention and home practice for SSD has been found previously (McAllister et al. 2011; Watts Pappas et al. 2016). Preliminary evidence in other disciplines suggests that it may be possible to teach the skills needed to foster positive therapeutic alliances to improve outcomes (Crits‐Christoph et al. 2006). Our study indicates that such training may be necessary for SLTs to ensure that parents experience effective therapeutic relationships with parents.

### 4.4 Summary

The COM‐B model was not discussed with our participants, and it is unclear whether the SLTs supporting our participants were aware of the model. Previous research indicates that SLTs have little understanding of how to effectively support parents of children with SSD (Sugden et al. 2018) and that SLTs may require postgraduate training to ensure their **capability** for working with parents (Harding et al. 2025; Pritchard et al. 2025). Our findings also suggest that parents who have recently attended an intervention for their child's SSD were expected to complete home practice without the SLT developing their **capability** or **motivation** sufficiently. We suggest that knowledge of the COM‐B model and the possible factors within the framework that drive parents’ **behaviours** would be valuable in supporting SLTs’ training to maximise **opportunities** for, **capability** of and **motivation** of parents. Explicit training for SLTs based around this framework, including embedding the framework into pre‐registration courses, could therefore support SLTs to successfully enable parents to deliver home practice effectively.

### 4.5 Areas for Future Research

The results of this study raise some potential areas for future investigation, particularly in relation to wider representation when gathering parental voices. The difficulties experienced with scheduling for the three participants (specifically the two fathers) who signed up and were then unable to participate highlight limitations around the representation of the sample. It is possible that those who found it more difficult to schedule an interview may also find it more difficult to schedule home practice due to their busy schedules. However, all these potential participants had indicated that they had completed home practice with their child, and so inclusion of their voices could have potentially added further insight into how they manage this and how their child's SLT facilitates it. Fathers are underrepresented in studies seeking to gather parental perspectives in the field of speech and language therapy. There are examples of studies where all participants are mothers (e.g., Sugden et al. 2019), where fathers are only included as part of mother–father dyads (Davies et al. 2017; Watts Pappas et al. 2016) or where most participants are mothers (e.g., in Markham and Dean 2006, where one out of 11 parents participating in focus groups were fathers). Including fathers may be particularly important as studies in other areas of speech and language therapy have found that there is a difference in how mothers and fathers experience and respond to SLT intervention (e.g., Kreiser and Segal 2025). Consideration of different methods of recruitment may be needed to recruit fathers to research studies. Without the inclusion of fathers in studies such as this, it is not possible to know how or if their experiences differ. Capturing fathers’ voices more widely in research of this nature, therefore, has the potential to raise new facilitators and barriers to completion of home practice that are yet unexplored.

Our participants mentioned a wide range of strategies that they felt supported them in their home practice for their children with SSD. However, it is unclear which or which combination of these strategies will have the most impact on home practice and thus the success of the intervention. Examining these more closely within existing theories of adult learning, considering the interaction of these strategies with a parent's learning style and across different SSD diagnoses, would add to our knowledge of what SLTs can do to support parents to deliver home practice effectively. The importance of individualisation and flexibility of approach may also be considered to support SLTs to know which strategy to use, with which parent and when.

There is emerging research that applies behaviour change techniques to interventions requiring home practice. For example, Barnett et al. (2023) identified and applied behaviour change techniques to parent‐led language interventions to clarify how SLTs use behaviour change techniques in clinical practice. Explicitly highlighting behaviour change techniques and models supports clinicians to purposefully apply these when coaching a parent to implement the intervention at home. This study further demonstrates that there is scope for behaviour change theories and models to be applied to other interventions, with a home practice component in the field of speech and language therapy.

### 4.6 Limitations

Whilst the inclusion of the voices of children was a strength of the project, only one parent was included in the PPIE activities that informed the design and delivery of the project. This involvement ensured that the study was relevant and accessible to parents, including the research questions and wording used throughout the study materials. However, working with more parents in a PPIE capacity for the duration of the project would have strengthened the design, delivery and potential impact of the project further. For example, involving parents during the analysis would have challenged our assumptions and views based on our own experiences. Including both parents and children in a PPIE capacity, for the duration of this interview study, would have further contributed to ensuring the interpretation and implications for practice were relevant to both parents and children.

All interviewers were qualified SLTs. Whilst every effort was made to ensure parents felt safe to share, and that the power within the relationship was balanced, it is possible some parents felt that the interviewer was in a position of power, influencing what parents felt comfortable sharing. Additionally, whilst EP did not have relationships with any of the parents prior to the study, she developed a relationship through the PPIE sessions with one participant whom she interviewed towards the end of the study. This existing relationship may have impacted what the parent shared.

Some parents had experienced interventions targeting both language and SSD, and at times found it hard to differentiate between speech and language therapy targeting language and speech. We mitigated this with frequent reminders that the focus was on speech, though it is possible some data we collected may not be directly relevant to speech intervention.

Finally, eight out of nine parents had the resources and capacity to seek alternative provision when they were unhappy with the statutory speech and language therapy that they had received, and their experiences may therefore be unique. However, this position allowed them to compare independent, university and NHS services, which may have strengthened their perceptions of what they need from their SLT.

## 5 Conclusion

This study highlights the complexity of supporting parents to become **capable** implementors of intervention. Home practice appears to work best for parents when SLTs spend time getting to know the parent and child, thereby developing positive relationships and individualising the service they provide. Parental perspectives indicate that **motivation** for home practice further increases when SLTs consider how parents understand SSD and use a multi‐modal approach to build understanding. To drive a shift in culture and practice for children with SSD, there appears to be a need to train SLTs in how to support parental **capability**, **motivation** and **opportunity** to deliver home practice. Utilising available resources in different ways and considering family contextual factors, such as sibling involvement more flexibly, to identify potential facilitators, may increase **opportunities** to support parents and may therefore support a move towards higher quality services for children with SSD.

## Funding

This study was funded by the University of Reading.

## Ethics Statement

Ethical approval was gained for the parent study from the University of Reading, Psychology and Clinical Language Sciences, School Research Ethics Committee 2023‐223‐EP. Ethical approval was gained for the PPIE work from the University of Reading, Psychology and Clinical Language Sciences, School Research Ethics Committee 2023‐224‐EP.

## Consent

Written and verbal consent was gained from participants of both the study and the PPIE group following the protocols approved by the ethics committee. The children in the PPIE group completed an assent form before each session to ensure they were happy to participate and to make sure they knew they could stop their involvement at any time. The consent form for the study and the PPIE, as well as the PPIE assent form, are available in the supporting materials.

## Conflicts of Interest

Author Dr Jill Titterington is editor‐in‐chief of the IJLCD.

## Supporting information




**Supporting File 1**: jlcd70280‐supp‐0001‐SuppMat.docx

**Supporting File 2**: jlcd70280‐supp‐0002‐SuppMat.docx

**Supporting File 3**: jlcd70280‐supp‐0003‐SuppMat.docx

**Supporting File 4**: jlcd70280‐supp‐0004‐SuppMat.docx

## Data Availability

Additional data are available in the supporting materials. Please contact the author for copies of the full anonymised transcripts.

## Endnote

1 […] indicates that some text has been removed. Text is removed when the participant added information that was not relevant to their point, such as an aside.
