# Supplementary material for: Parental Perspectives and Experiences of Working With Speech and Language Therapists to Support Home Practice for Their Child With a Speech Sound Disorder: A Qualitative Study
Source: Int J Lang Commun Disord. 2026 Jun 22;61(4):e70280. doi: 10.1111/1460-6984.70280 (PMC13288021; doi:10.1111/1460-6984.70280)
Supplement: Supplementary file 1 — Supporting File 1: jlcd70280‐supp‐0001‐SuppMat.docx [file JLCD-61-0-s004.docx]

# Appendix 1 - semi-structured parent interview topic guide

### **KEY**

**Research questions**

Core questions for the parents

*Possible probe questions (used based on parent’s responses)*

*How the topic guide is informed by previous research (xx xx 2024, 2026)*

*How the topic guide has been informed by PPIE activity.*

*How questions are guided by relevant theory/frameworks*

Questions were used flexibly. Probe questions were asked to explore each area dependent on the participants’ responses.

*N.b. the topic guide is structured around four research questions, during the analysis questions 2 & 3 were collapsed due to considerable overlap. Adjustment of research questions of this nature is acceptable in reflexive thematic analysis (Braun & Clarke, 2021)*

## Introduction

## Opener

Show session set up photos (figure 2 in paper)

***Describe how the sessions with your Speech and Language Therapist are set up?***

*Environmental factors, such as how the room is set up can ensure opportunities are provided and impact parental motivation and relationship building. Children indicated the different ways in which the sessions are set up with their parents in different roles.*

**Section 1 (aims to address research question - What is the experience of parents/primary carers of children with speech sound disorder of working with SLTs within direct therapy sessions for their child?)**

- What has been/is your role in your child’s speech and language therapy?

*What is the SLTs role? What did you expect your role to be before the first session? Have your expectations of these roles changed over time? how important is it that the SLT discusses things with you vs works with your child directly? What are your experiences of the SLT discussing things with you, does this support your understanding? what guidance did you have from the SLT about what to do in the sessions?*

*Parents play an important role in intervention but are not always clear what their role is. SLTs do not always agree what a parent’s role is. SLT and parent expectations of roles can differ. Most children liked that their parents joined in with the session. Parents’ actively joining in can facilitate their learning and provide opportunities.*

**Section 2 (aims to address question** - **Which strategies do parents/primary carers think work well to build the relationship between parents/primary carers and SLTs?)**

- What do you think are the relationships that matter when receiving SLT? (SLT/child, child/parent, SLT/parent, between all 3)

*Has it changed over time? What is your child’s relationship with the SLT like? Is this important to you, why, what about (the wider family, SLT/child, SLT/parent, child/parent)? What worked well to build the relationships? Have you always seen the same SLT? If not, does this matter?*

*Therapeutic relationships between stakeholders (including SLT, child, parent and other people in a child’s life) are important to the success of intervention and are intertwined. Relationships are a motivating factor. Children talk positively of their SLT saying they like them, this will explore what SLTs might do to ensure this happens. Some children indicated that they didn’t like their parents and the SLT talking.*

- What are important qualities in an effective SLT?

*Why/how does this support you and your child? How do these qualities help with the home practice?*

*This has not been explored in detail this question looks to explore this subject further. Children have indicated that SLTs help them. This will address motivation and developing parental capability.*

## **Section 3 (aims to address question - Which strategies do parents/primary carers think work well to support parents/primary carers to deliver home-practice effectively with their child at home?)**

- How did the SLT work with you in the sessions so that you understood what to do at home?

*Did you have a go at the home tasks in the sessions? Did the SLT try to understand your parenting style? Do you think this is important for successful home practice? Did they discuss what was possible for you and your family? Did they explain why home practice was required? Were you given guidance/advice about how often/how long to practice for? What is the most useful piece of knowledge/advice that your SLT has given you that has supported you to understand your child’s SSD? Do you prefer structured activities or those which are flexible to fit into your daily routine? Do you feel confident working with your child at home? how led by the child is your SLT?*

*Adults learn best when a diverse range of approaches to coaching are taken. Developing parents’ knowledge of intervention, including the why and the how is important to parents and SLTs. This supports parental capacity. Understanding SLT’s parenting styles and family routines influences how successful home practice is. Children spoke of fun and games being important to engagement in sessions.*

- Has your confidence or capability changed over time and if so why?

Share rating from children when asked ‘when it’s time to practice how do you feel?’

*Was there anything that happened in the first few sessions that you think made the difference? What could the SLT have done to support your confidence and capability from the beginning? What did the therapist do that allowed your confidence/capability to grow or not? Is your confidence linked with your child’s progress? Does your SLT support you to celebrate things that are going well? how led by your child is your home practice?*

*Parents’ confidence and capability changes over time. This question looks to explore what happens supports a parent to feel capable. Children discussed how their parents helped with their speech at home like their SLT did in the session.*

## **Section 4 (aims to address question - What do parents/primary carers of children with speech sound disorder think are the barriers and facilitators to working with their child effectively at home?)**

- What motivates/encourages you to work with your child, on their speech at home?

*What supports you to feel motivated to complete therapy with your child? How easy is it to work with your child at home? Why/why not? How important is fun?*

- *What stops you/might stop you working well with your child at home?*

*Is there anything that helps with this? Is home practise ever difficult? If so why? Is there anything your SLT could do to help you when it’s difficult? Was any written information clear to you?*

*SLTs attitudes and behaviours can impact how they approach parent coaching, this could also impact parents’ experiences and perceptions. Intertwined relationships may mean that SLT, child or parent factors provide barriers or facilitators. Exploring motivation and opportunities for behaviour explicitly with parents. Children like working with their parents at home when fun and games are included. Some children spoke about what their home practice included, how their parents supported this, including monitoring sheets, number of times they needed to say something. Siblings were also mentioned by several children both as a barrier to home practice and a facilitator, this will be explored with parents.*

**Summary**

- **Any final thoughts or comments before we close?**

Thank participants for coming, let them know about the next phases of the project and how this study will contribute.
