# Supplementary material for: Parental Perspectives and Experiences of Working With Speech and Language Therapists to Support Home Practice for Their Child With a Speech Sound Disorder: A Qualitative Study
Source: Int J Lang Commun Disord. 2026 Jun 22;61(4):e70280. doi: 10.1111/1460-6984.70280 (PMC13288021; doi:10.1111/1460-6984.70280)
Supplement: Supplementary file 2 — Supporting File 2: jlcd70280‐supp‐0002‐SuppMat.docx [file JLCD-61-0-s003.docx]

# Appendix 2 – First author’s reflexivity and positionality

All sessions were conducted by myself or EP, both SLTs with nearly 20 years' experience, I am also a PhD student and lecturer with a specialist interest in SSD. EP is an experienced researcher. JT is an SLT and VS a clinical linguist who are both experienced researchers with experience of qualitative research. Myself, EP and JT all have experience working with children with SSD and their parents and were aware that we have our own opinions of what works well in the context of supporting parents from our clinical experiences. Fortnightly discussions with the whole team were important to ensure rigor with VS acting as a non-SLT, critical friend and thus enhancing my insight during the study. These discussions included reflections on the role that our experience and our presence had on the data generation, the analysis and the final interpretation. For example, where parents reported experiencing services that didn’t fit current evidence-base practice we discussed my initial reaction to this and then the impact that my knowledge had on the analysis of the data, such as focusing on more negative interpretations in these instances in light of my knowledge of the impact of this. In a previous study with SLTs one of the assumptions around independent SLTs being able to offer more than NHS SLTs had been challenged, thus making us more open to hearing positive experiences of NHS service provision. However the parents we spoke to had mostly felt that the frequency of service they received from the NHS was not enough.

Focus groups allowed parents to expand on each other’s ideas and experiences and compare them to their own, whereas in the interviews participants only had their own experiences to draw on. However, the 1:1 nature of the interviews allowed parents to reflect on what was most pertinent to their experiences and not be drawn to discuss things less relevant to them. Allowing them to reflect on potentially difficult experiences in a more confidential environment. The online environment also had an impact on our data generation. It was harder for participants in the groups to interject and/or respond in the way they may have done in a face-to-face setting. This was less of an issue in the individual interviews where it was much clearer whose turn it was to speak. I kept a reflective diary throughout the process to detail this.
