# Supplementary material for: Parental Perspectives and Experiences of Working With Speech and Language Therapists to Support Home Practice for Their Child With a Speech Sound Disorder: A Qualitative Study
Source: Int J Lang Commun Disord. 2026 Jun 22;61(4):e70280. doi: 10.1111/1460-6984.70280 (PMC13288021; doi:10.1111/1460-6984.70280)
Supplement: Supplementary file 3 — Supporting File 3: jlcd70280‐supp‐0003‐SuppMat.docx [file JLCD-61-0-s001.docx]

# Appendix 3 – semantic and latent code examples

| **Data extract** | **code** | **notes** |
| --- | --- | --- |
| **Semantic codes** | | |
| ‘his brother has clubs. So that makes it difficult.’ P8 | Conflicting demands can create a barrier to parental involvement | Here the parent explicitly comments on a conflicting demand that stops her working well at home. |
| ‘I have to be careful with games because he will get very, very distracted. Sometimes, it might be better to just sort of go straight in and do the practice for him.’ | Games are not always supportive of practice | Here P1 explicitly discusses how games do not always support the home practice. |
| **Latent codes** | | |
| ‘I did sometimes think, oh, I wonder what it would be like if I wasn't there at all. (P9) | Children respond differently to different people and this can impact parental involvement in session and at home. | Here P9 discusses the possibility of her not being involved in the session and wonders what it would be like for her daughter. The implication here is that her daughter would respond differently (better?) to the SLT without her parent. This potentially impacted the session and may also impact the sessions led by her at home as the parent wouldn’t have the opportunity to learn if they were absent. |
| ‘I wasn't consistent on all these tasks and I'm a structured person and I love the guidance, but I couldn't do it. So you felt a bit like you're the bad student or the, you know’ (P2) | Parents can feel pressure and guilt to complete home practice | Whilst P2 does not explicitly talk about guilt, the reference to being a bad student indicates that she feels guilty or judged in some way for not doing the homework. |
