# Supplementary material for: Parental Perspectives and Experiences of Working With Speech and Language Therapists to Support Home Practice for Their Child With a Speech Sound Disorder: A Qualitative Study
Source: Int J Lang Commun Disord. 2026 Jun 22;61(4):e70280. doi: 10.1111/1460-6984.70280 (PMC13288021; doi:10.1111/1460-6984.70280)
Supplement: Supplementary file 4 — Supporting File 4: jlcd70280‐supp‐0004‐SuppMat.docx [file JLCD-61-0-s002.docx]

# Appendix 4- Examples of how PPIE activity influenced the research

Methods were repeated and topics revisited across the sessions in line with recommendations for working with children to ensure accurate understanding of contributions (Clark, 2017).

| **PPIE activity** | **Associated image** | **Impact on research** |
| --- | --- | --- |
| Using toys to role play a typical intervention session with their SLT | 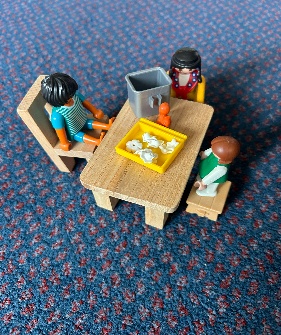 | Provided insight into how parents are involved in the sessions and the relationships between SLT, parent and child. Images from this were used in **interview slides** (see figure 2) to trigger discussion about the physical environment and the impact this has on parents’ involvement within the sessions and the impact this then has on home practice. |
| Responding to statements to explore the relevance and importance of simplified versions of the **codes** and **initial candidate themes** | 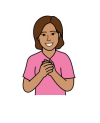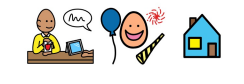Parents and children like their SLT | In this example the **candidate theme** being explored is ‘Successful relationship building is foundational to successful home practice.’ Children in PPIE group rated this with high importance and talked of why they liked their SLT. This suggested that it was important to continue to develop the analysis around this candidate theme and explore the potential impact this finding would have on clinical practice. |
| Drawings of the child working at home on speech | 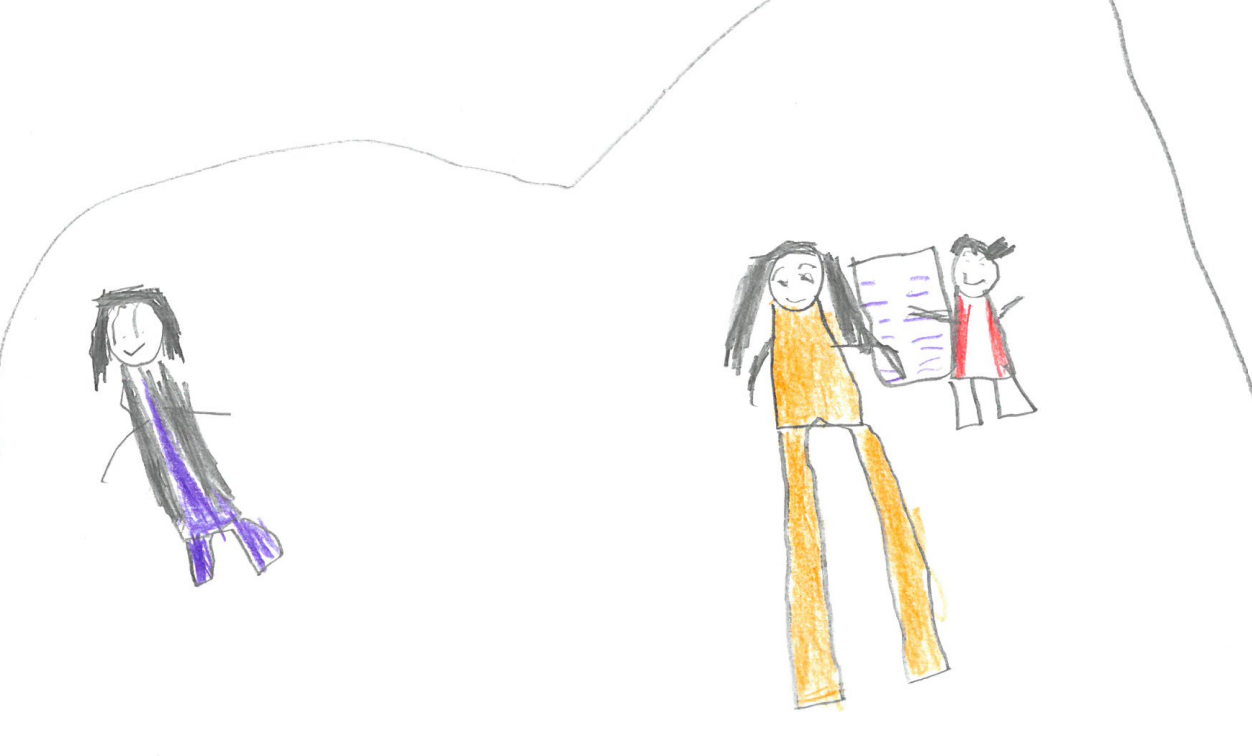 | Children’s drawings drew out the role of monitoring and rewards in home practice. For example, in this image the child is ticking his productions off, informing questions in the **topic guide** around how parents keep track of home practice, feedback to SLT and dose in home practice. |
| Ranking of SLT activities | 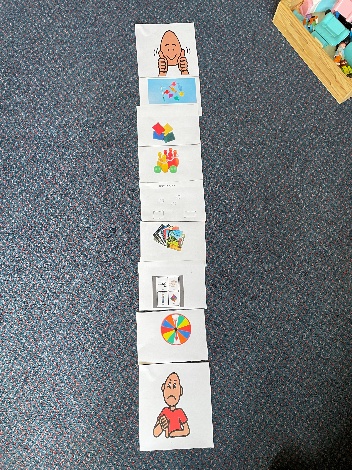 | Children selected games and activities that they had completed in their SLT sessions and ranked them from those they liked to those they did not. This led to discussions with the children about what they did and did not like about the games and if these were used at home. This led to refinement of the **topic guide** to include how the SLT supported parents to incorporate games from the sessions into home practice. |
| Reviewing video clips of themselves and their parent completing home practice, and rating how they felt during session | 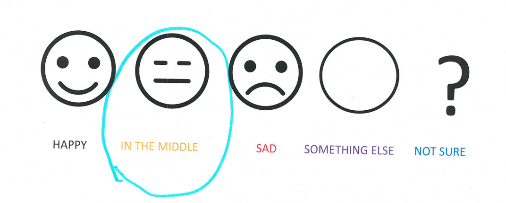 | Children commented on the games and activities that they had completed in the session. Across the children a range of games and activities were seen, including hide and seek, winning tokens, using stampers. All the children commented on the game they were playing being fun. This led to inclusion of questions in the **topic guide** around the importance of fun and games to motivate children and parents. Children spoke of a range of reasons for liking/not liking home practice. The role of siblings in home practice was raised by several children and led to the inclusion of questions about extended family in home practice. Selection of rating scales were used in the **interview slides** to trigger discussions around child’s enjoyment of home practice.  Rating scales and quotes from the children about their ratings were used in the **research output video** to illustrate what is important to children when completing home practice. |
| Taking photos – children were given an ipad and asked to explore the clinic space and take photos demonstrating what is important to their SLT. | 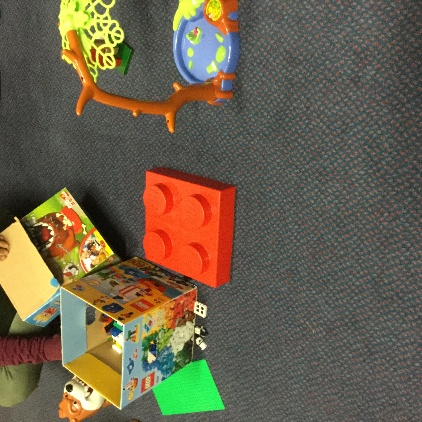 | These photos were combined with other resources from the children such as quotes, rating scales and used in the **research output video**. Discussion, supported by pictures, also contributed to the output being in video form and with children’s voices as the children felt strongly that the video should be voiced by a child. |

Clark, A. (2017). *Listening to young children: a guide to understanding and using the mosaic approach* (3rd ed.). Jessica Kingsley Publishers.
